# 2209. Reducing Inappropriate Antibiotic Prescribing for the Treatment of Urinary Tract Infections (UTI) in Urgent Care Clinics

**DOI:** 10.1093/ofid/ofad500.1831

**Published:** 2023-11-27

**Authors:** Christine A Vu, Veronica Salazar, Fenil Patel, Lilian M Abbo, Rossana Rosa

**Affiliations:** Jackson Memorial Hospital, Miami, Florida; Jackson Memorial Hospital, Miami, Florida; Nova Southeastern University College of Pharmacy, Miami, Florida; University of Miami Miller School of Medicine, Miami Transplant Institute and Jackson Health System, Miami, FL; Jackson Memorial Hospital, Miami, Florida

## Abstract

**Background:**

In 2020, The Joint Commission released new standards to promote expansion of antimicrobial stewardship into the ambulatory care setting. Urinary tract infections (UTIs) are one of the most common bacterial infections encountered in the outpatient setting and therefore an opportunistic area for intervention. The aim of our study was to evaluate current outpatient prescribing patterns for the treatment of UTIs and develop a methodology to improve antibiotic prescribing.

**Methods:**

A single-center, retrospective study was conducted across 5 Jackson Health-University of Miami Urgent Care Clinics (UCC) in Miami, Florida. We utilized ICD-10 diagnosis codes to identify adults diagnosed with UTIs between January 1, 2021 and October 31, 2022. We identified the top five prescribers and audited 10% of their prescriptions. The data was analyzed and as our intervention, we shared results with UCC leadership and disseminated provider-specific report cards to highlight inappropriate prescribing practices. After this, we allowed 3 months for change to occur and set a goal of reducing the percentage of inappropriate UTI durations by 50%.

**Results:**

For baseline data, 123 charts were reviewed. We identified 95 patients with cystitis (77.3%), 26 pyelonephritis (21.1%), and 2 asymptomatic bacteriuria (1.6%). Inappropriate prescribing ranged from 17.6% to 80.6% between the UCC prescribers (Figure 1). Various types of prescribing opportunities were identified, with the most common being prolonged length of therapy (Figure 2).

For post-intervention, 114 charts were reviewed. We identified 90 patients with cystitis (78.9%), 22 pyelonephritis (19.3%), and 2 asymptomatic bacteriuria (1.8%). Inappropriate durations of UTI treatment were reduced by 25% (Figure 3), with the greatest improvement seen in nitrofurantoin prescribing (Figure 4).

Figure 1

**Figure:**
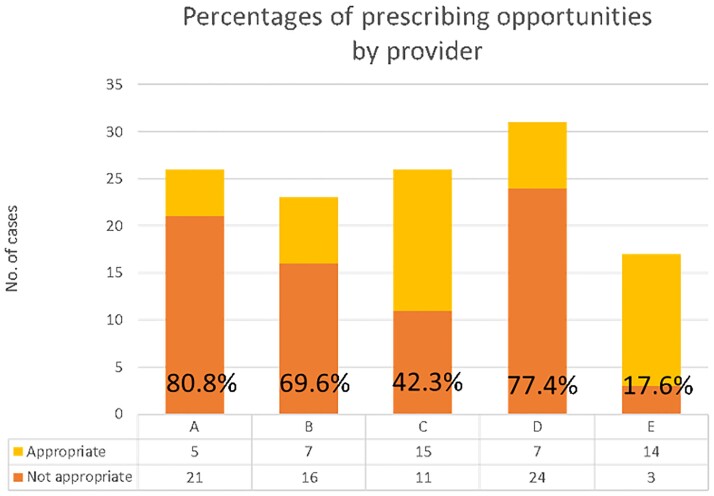

Figure 2

**Figure:**
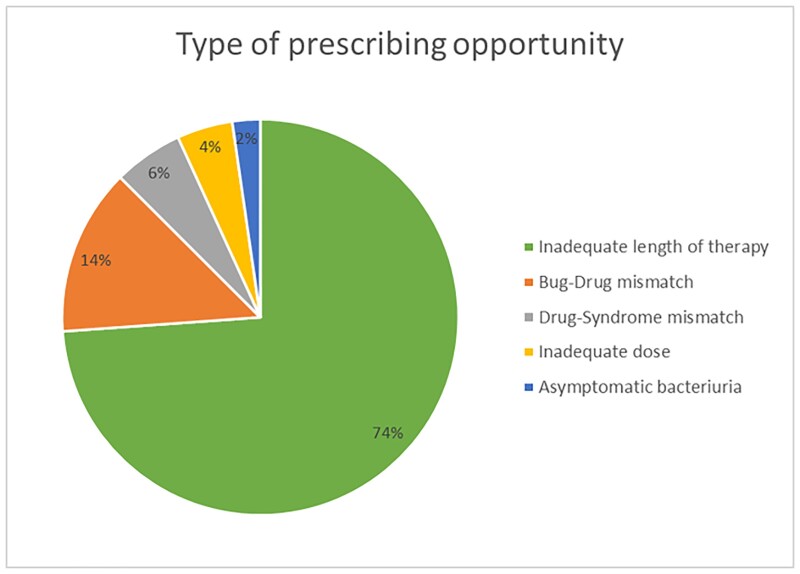

Figure 3

**Figure:**
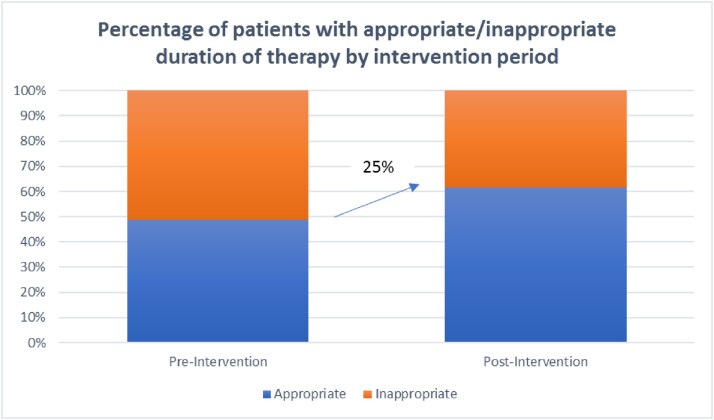

**Conclusion:**

Retrospective audit and feedback using provider-specific report cards was an effective antimicrobial stewardship strategy for the ambulatory care setting. Our intervention led to reduced durations of UTI treatment for nitrofurantoin but not necessarily with fluoroquinolones, trimethoprim/sulfamethoxazole, or beta-lactams. Next steps will include providing more education to further promote appropriate use of all classes of antibiotics.

Figure 4

**Figure:**
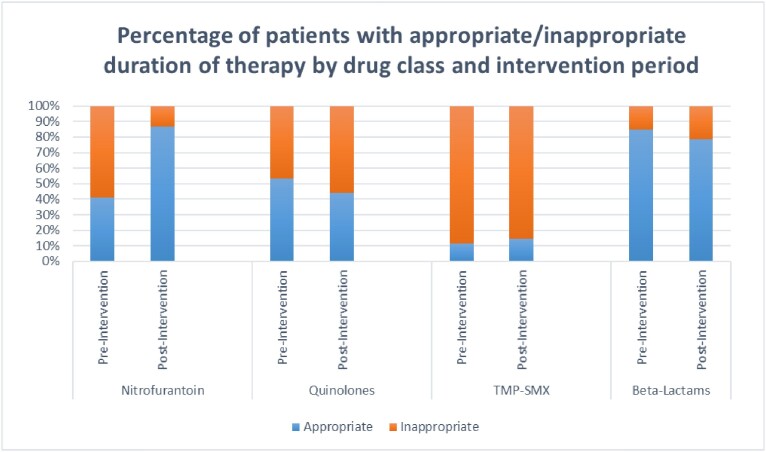

**Disclosures:**

**Lilian M. Abbo, MD, MBA**, Ferring: Advisor/Consultant|Pfizer: Advisor/Consultant|Regeneron: Grant/Research Support|Shionogi: Advisor/Consultant

